# Psychological restoration depends on curiosity, motivation, and species richness during a guided bird walk in a suburban blue space

**DOI:** 10.3389/fpsyg.2023.1176202

**Published:** 2023-06-05

**Authors:** Christoph Randler, Janina Vanhöfen, Talia Härtel, Freya Neunhoeffer, Cheyenne Engeser, Christian Fischer

**Affiliations:** ^1^Department of Biology, University of Tübingen, Tübingen, Germany; ^2^Hector Research Institute of Education Sciences and Psychology, University of Tübingen, Tübingen, Germany

**Keywords:** psychological restoration, personality, bird species knowledge, big five, species richness, recovery

## Abstract

Urban and suburban green and blue spaces are important places for human recreation, and the impact of biodiversity on psychological and recalled restoration has received much attention. This study addresses the relationship between bird species richness and restoration in a controlled field experiment (guided bird walk) applying a battery of individual trait scales (need for cognition, personality) as predictors of restoration. We found a significant positive relationship between the number of bird species present and recalled restoration. Personality, bird species knowledge, bird related interest as test measures, demographics and birding specialization as self-report had no influence on psychological restoration. However, need for cognition correlated positively with psychological restoration, thus providing a new predictive variable. All subscales of the intrinsic motivation scale (enjoyment, perceived competence, perceived choice, pressure/tension) were positively correlated with restoration except of pressure/tension. Learning emotions like interest and well-being were positively related to restoration, while boredom was negatively related. Therefore, we suggest research to examine the restorative function of more cognitive-oriented programs because people may also need cognition when it comes to restoration. We also suggest a broader focus on education and cognitive aspects when it comes to linking biodiversity and health within the framework of ecosystem services.

## 1. Introduction

Urban and suburban green and blue spaces are important places for human recreation, and especially the impact of biodiversity on psychological restoration, well-being and physical restoration has received much attention during the last decades (e.g., Dallimer et al., 2012; Braubach et al., 2021; Fisher et al., 2021). However, some results are still inconclusive, and individual variation, for instance related to personality characteristics, has been less studied. There are differences between green spaces in relation to their restoration benefits (Wood et al., 2018). We here addressed this by using standardized field trips to the same location with a focus on birds, to assess the influence of the actual bird diversity and various individual variables on the outcome of psychological restoration. Psychological restoration was the main focus of this study. Although physical restoration is another important aspect it was not considered here. The field trip was designed in the form of a guided bird walk (Tryjanowski et al., 2022). This study hopes to inform researchers when studying restorative function, as well as stakeholders working in the intersection of biodiversity and health.

## 2. Theoretical background

### 2.1. Biodiversity and psychological restoration

Two main theories are related to psychological well-being and nature. Following the Attention Restoration Theory (ART), spending time in nature allows recovering from attention fatigue (Kaplan and Kaplan, 1989; Kaplan, 1995). The stimuli provided by nature should be of not too high, but also not too low impact to allow attention to be restored. According to the Stress Reduction Theory (SRT; Ulrich, 1983), high levels of biodiversity could also benefit psychophysiological stress recovery. Therefore, both theories predict that a stay in nature improves psychological health and well-being. People experience nature and therefore biodiversity even in small urban green spaces (White et al., 2019; Wyles et al., 2019). In addition to positive effects on human health and well-being, contact with nature can support psychological restoration (Ulrich, 1983; Kaplan and Kaplan, 1989; Kaplan, 1995; Sandifer et al., 2015; White et al., 2019; Fisher et al., 2021). Therefore, contact with nature means replenishing cognitive resources and reducing stress (Ulrich, 1983; Kaplan and Kaplan, 1989). However, it may not be only nature itself, but rather the experience of biodiversity that positively impacts well-being (Dallimer et al., 2012). Some previous studies already provide evidence for the relationship between biodiversity and health. For example, Lindemann-Matthies and Matthies (2018) showed stress reduction by measuring systolic blood pressure after viewing plant diversity. In addition, Lindemann-Matthies et al. (2010) reported that people valued a high plant species richness more than a few plant species. Concerning different measures of biodiversity, Fuller et al. (2007) found a positive relationship between bird diversity and well-being, and Dallimer et al. (2012) proposed an association between perceived biodiversity and well-being.

### 2.2. Bird diversity and psychological restoration

In addition to nature itself, birds may play a relevant role in health and well-being. For example, Methorst et al. (2021) showed that bird diversity is related to well-being, and neighborhood satisfaction is related to bird diversity (Hepburn et al., 2020). People felt happier in a more bird-rich environment (Cameron et al., 2020). Concerning birdsongs, some studies reported a positive effect of birdsongs on human health and well-being (Stobbe et al., 2022). A higher birdsong or acoustic diversity was rated as more pleasant (Hedblom et al., 2014), and birdsong lowered anxiety and negative affect (Fisher et al., 2021; Stobbe et al., 2022), and, in turn positively influenced well-being (Fisher et al., 2021) and perceived restoration (Ferraro et al., 2020; Zhu et al., 2020). Many, if not all studies, found no relationship between knowledge and perceived or recalled restoration (Dallimer et al., 2012). This is in line with the Attention Restoration Theory that suggests that people can relax and restore without any detailed knowledge about species, nature and biodiversity.

### 2.3. Field trips and guided bird walks as a source of restoration

Although it seems established that bird species richness impacts health and well-being, there are few studies controlling more directly the experience of the participants, for instance, by standardizing the environment and by standardizing the time spent in nature. Murawiec et al. (2021) developed a therapeutic bird walk during the COVID-19 pandemic to foster mental health (Tryjanowski et al., 2022). Such a therapeutic walk focuses more on the experience and enjoyment of nature but is still connected with watching birds. It is therefore different from the usual birding activities carried out by more specialized and committed birders (*cf.* Randler and Großmann, 2022). In this context, we assume that a slower-paced field trip would be beneficial in contrast to usual birding field trips.

### 2.4. Individual factors related to psychological restoration

Individual factors can be assessed on the state (situational) and the trait level. Situational variables are fluctuating during the day or in settings, such as a sense of accomplishment (Gläser-Zikuda et al., 2005). Trait characteristics refer to enduring variables that show no or low variation across situations, e.g., personality.

#### 2.4.1. Personality

Individual differences have been a blind spot in epidemiological research on green space and health (Feng et al., 2022), especially to what extent the benefits are related to personality. Few studies address the ‘big five’ personality traits (conscientiousness, agreeableness, neuroticism, openness to experience, and extraversion). Feng et al. (2022) reported that girls with low levels of extraversion and high levels of neuroticism may potentially benefit more from high-quality urban green spaces in their direct environment. In a study using a virtual environment, the extraversion dimension was weakly and negatively related to the fascination and being-away change score as a dimension of restorativeness. Other personality dimensions were unrelated (Senese et al., 2020). Another study suggested that individuals who report lower levels of neuroticism glean noticeably greater psychological benefits from greenspace (Ambrey and Cartlidge, 2017). In a survey study among five countries, however, personality domains of the big five have been unrelated to restoration (Subiza-Pérez et al., 2021). Thus, the evidence concerning the relationship between personality and psychological restoration remains inconclusive.

#### 2.4.2. Need for cognition

The need for cognition refers to an individual’s motivation to engage in and enjoy thinking (Cacioppo et al., 1996). It is a personality trait that describes the extent to which a person values and seeks out opportunities for mental engagement and complex problem-solving. People with a high need for cognition are often curious, enjoy challenges and seek out new information, while those with a low need for cognition tend to avoid mental effort and prefer simpler activities (Wu et al., 2014). The construct of the need for cognition was related to achievements, especially academic ones (Colling et al., 2022).

#### 2.4.3. Situational variables emotions and motivation

Emotions and motivation are fluctuating across situations (Gläser-Zikuda et al., 2005; Wilde et al., 2009). For example, a person can be generally uninterested in a subject, (e.g., biology), but during a given situation (e.g., an encounter with a fascinating animal), the situational interest may be high, but the general interest (in biology) may not change (Randler and Bogner, 2007). This can be similarly applied to intrinsic motivation during a learning process. Both concepts have been widely used in previous research on learning motivation and intrinsic motivation and questionnaires were established and validated to address and measure fluctuations in these variables (Gläser-Zikuda et al., 2005; Wilde et al., 2009; Randler et al., 2011). Learning emotions are related to the key variables interest, well-being, and boredom (Gläser-Zikuda et al., 2005). This has been established in many different school- and university-based studies and many studies report correlations between these emotions and academic achievement, in a way that interest and well-being were positively related to educational outcomes, and boredom negatively (Gläser-Zikuda et al., 2005; Wilde et al., 2009; Randler et al., 2011). Intrinsic motivation is based on self-determination theory (Ryan and Deci, 2017), which highlights the roles of experiencing autonomy, competence, and social relatedness. Concerning learning situations, four dimensions are usually addressed: enjoyment, perceived competence, perceived choice, and pressure/tension (Wilde et al., 2009). Similarly, learning emotions, enjoyment, autonomy, and competence are positively related to educational outcomes, while pressure/tension negatively influence achievement (Wüst-Ackermann et al., 2018).

Both concepts have been largely applied in the learning sciences. However, to our knowledge, they have never before been linked with psychological restoration. As our experimental study had a learning component, i.e., the participants learned about the birds they identified in a self-directed, autonomous manner, we feel that the application of these measures will help to improve our understanding of the relationship between learning emotions, motivation, and psychological restoration.

### 2.5. The current study

This study assesses both the individual factors that might be relevant for psychological restoration and whether bird species richness is related to individual psychological restoration. Further, we study the relationship between personality and psychological restoration. In contrast to previous studies, we measured the restoration under controlled environments in an experimental design and applied a variety of questionnaire scales. As some scales (developed as trait measures) have been applied prior to the field trip, a causal relationship may be supposed. We applied bird species knowledge, as well as personality and individual traits (need for cognition) prior to the field trip to use these measurements as predictors of the restorative outcomes. Thus, this research fills the gap in our knowledge by providing a field trip under controlled supervision, in combination with an assessment of the bird diversity/species richness exactly at the same location during the same time frame, and finally, an assessment of personality and motivation and learning emotions.

Our research questions (RQs) are as follows:

RQ1: Is there a relationship between bird species richness and psychological restoration?
RQ2: Is personality related to psychological restoration?
RQ3: Is knowledge about and interest in birds related to psychological restoration?
RQ: Are emotions, motivation and need for cognition associated with psychological restoration?

## 3. Materials and methods

### 3.1. Study site Hirschauer Baggersee

The Hirschauer Baggersee is a suburban blue space, located near the village Hirschau, Tübingen SW Germany. Data from eBird shows that it is a hotspot with 95 different bird species reported so far, which is a relatively high number.^1^ Further, the bird species at this suburban location are relatively tame and easy to observe because they are habituated to human presence. In comparison to other recreational places in the same area, the flight initiation distances of birds at Hirschauer Baggersee are lower (Reichert, 2022; unpublished B.Sc. thesis). Finally, the location contains both blue and green spaces and therefore, waterbirds and forest birds can be observed simultaneously.

### 3.2. Guided birding activity

The birding field trip was situated in a pretest-posttest design with tests carried out at the university campus. After the pretest, the participants went by bus to the birding location. The field trip lasted about 2 h during the morning. We carried out five different field trip days between July 6, 2022, and July 19, 2022 with group size ranging from 20 to 32 participants. All field trips led to the exact same location and the same route was used, spending the same amount of time in the field. The field trips were led by the first author and assisted by members of the research team. Weather conditions were stable during this period with the daily average temperature between 18.3 and 26.6°C and a peak temperature between 24.9 and 35.7°C. The weather was sunny without any rain, no wind, and with good sight conditions (Agrarmeteorologie Unterjesingen).^1^

### 3.3. Bird census counts

During the guided field trips, all bird species seen and heard, as well as their numbers, were recorded by an experienced field ornithologist (the first author). The total number of bird species ranged between 35 and 43. The counts are deposited in eBird in the respective checklists (see Appendix 1).

### 3.4. Participants and data collection

Participants were recruited via the mailing list of a large public research university in Southern German that reached about 30,000 potential participants. Participation was open for all enrolled students independent of major, age, or date of enrolment. A total of 132 students provided data for the restoration items (38 men, 90 women, 1 diverse, 3 did not provide data on gender). On average, students were 23.8 years old, SD = 3.4. Prior to the birding activity, a pretest was carried out at the university, measuring the following variables: bird knowledge test, birding specialization, bird-related interest, need for cognition, and personality (described below). Afterwards, the group went to the study site by bus. During the five field trips, the same route was always walked in groups together with experts of the research team. The participants were asked to identify birds independently with binoculars provided to them. The experts motivated the participants during the identification of the different bird species and assisted by using the scaffolding method. After the field trip, participants went back to the university by bus again and the posttest was conducted, measuring the following variables: intrinsic motivation, learning emotions, sociodemographic variables (described below). Each participant was only allowed to participate in a single field trip. As an incentive, course credits or alternatively, an identification book was offered after the completion of the study.

### 3.5. Questionnaires

#### 3.5.1. Psychological restoration

The psychological restoration scale consisted of two dimensions: recalled restoration and psychological restoration. We measured recalled restoration with a single item on a five-point Likert scale that asked how rested participants felt after having participated in the birding field trip with the choices “less rested than before,” “neither more nor less rested,” “a little more rested,” “more rested,” “much more rested” than before (von Lindern et al., 2013; Young et al., 2020). The “do not know” category was offered and coded as missing afterwards. The mean score was 3.00 (*SD* = 1.26). In addition, we used a three-item measure for psychological restoration adapted and slightly changed from Wyles et al. (2019), Nikunen and Korpela (2009), and Haugan (2015). The items were “I feel calm and relaxed,” “I feel refreshed,” and “I feel peaceful” and rated on a Likert scale from 1 (strongly disagree) to 5 (strongly agree). The Cronbach’s alpha of the three-item measure was 0.72. Mean scores were calculated for the scale (*M* = 3.52, *SD* = 0.87).

#### 3.5.2. Bird species knowledge and specialization

We used the bird species knowledge test following the methods of Vanhöfen et al. (2022). This test asked participants to identify 20 bird species by visual traits and 5 by auditive traits. These bird species identification items were scored according to a partial credit model with the correct identification receiving the value of 1.0, and partial credit with the value of 0.5. Incorrect answers received the value of 0.0. For example, the mallard (*Anas platyrhynchos*) was scored 1.0, whereas the description of this species as “duck” received 0.5 points. The raw scores for every bird species were summed up to a total identification score representing bird species knowledge (*M* = 11.94, *SD* = 4.66; Cronbach’s alpha 0.89). In addition to the species knowledge, birding specialization was measured with the subscale skill/knowledge out of Randler et al. (2022b). This scale asks for a self-assessment with three items, the number of bird species a person can identify without a book or app, (a) by sight (< 25, 26–45, 46–100, 101–250, 251–500, > 500) and (b) by sound (< 5, 6–10, 11–25, 26–80, 81–150, > 150), as well as a further self-assessment of one’s birding ability ranging from novice to expert on a five-point Likert scale. Cronbach’s alpha was 0.85 (*M* = 1.48, *SD* = 0.67).

#### 3.5.3. Big five personality

We used the German version of the big five Personality Inventory (BFI, Rammstedt and John, 2007). The BFI-10 consists of 10 items, two for each dimension of personality (Neuroticism, Extraversion, Openness, Agreeableness, Conscientiousness). Each of the dimensions is represented by a positive and a negative item (recoded). Participants responses were collected by a five-point rating scale from “does not apply at all” to “applies completely.” This is a time economic scale to measure the five personality dimensions with two items per domain. Mean scores were calculated for the scales and were *M* = 3.46 (*SD* = 1.03) for extraversion, *M* = 3.55 (*SD* = 0.98) for agreeableness, *M* = 4.00 (*SD* = 0.79) for conscientiousness, *M* = 3.26 (*SD* = 1.05) for neuroticism, and *M* = 4.04 (*SD* = 0.89) for openness.

#### 3.5.4. Intrinsic motivation

We used the short scale for intrinsic motivation (KIM, Kurzskala zur intrinsischen Motivation; Wilde et al. (2009)). This short scale is an adapted, time-economic version of the “Intrinsic Motivation Inventory” by Ryan and Deci (2017). The scale contains the factors enjoyment, perceived competence, perceived choice, and pressure/tension with three items each. Many studies have used the scale, and a confirmatory factor analysis recently confirmed the postulated four-factor structure in a large sample (*N* > 1,800; Wüst-Ackermann et al., 2018). Mean scores were calculated for the scales and were *M* = 4.09 (*SD* = 0.88) for enjoyment. *M* = 3.11 (*SD* = 0.84) for perceived competence, *M* = 3.86 (*SD* = 0.78) for perceived choice and *M* = 1.68 (*SD* = 0.68) for pressure/tension. Cronbach’s alpha was (in brackets): enjoyment (0.93), perceived choice (0.83), perceived competence (0.84), and pressure/tension (0.54).

#### 3.5.5. Learning emotions

Emotional variables were assessed with a scale provided by Randler et al. (2011), covering the three emotions interest, well-being, and boredom. Each construct was measured with three items on a five-point Likert scale from “fully agree” to “fully disagree.” None of the items were reverse coded. Mean scores were calculated for the scales and were *M* = 3.99 (*SD* = 0.90) for interest, *M* = 4.24 (*SD* = 0.80) or well-being, and *M* = 1.95 (*SD* = 0.88) for boredom. Cronbach’s alpha was (in brackets): interest (0.77), boredom (0.77), well-being (0.90).

#### 3.5.6. Bird-related interest

Bird interest and bird-related activities were measured with five items, coded from 1 to 5. The items were “*I am interested in ornithology/bird science*,” “*How often do you read about birds*?,” “*How often do you observe birds in nature*,” “*How often do you watch documentaries about birds*,” and “*The topic is important for me*” (adapted from Hummel et al., 2015). Cronbach’s alpha was 0.85. Mean scores were calculated for the scales (*M* = 2.95, *SD* = 0.81).

#### 3.5.7. Need for cognition

Need for cognition was measured with the short scale developed by Beißert et al. (2014). This is a four-item scale, coded on a 7-point ordinal scale. For further analysis we calculated the mean score across all items to generate an overall measure (*M* = 4.72, *SD* = 1.01). Cronbach’s alpha was 0.58.

#### 3.5.8. Demographic variables

Gender, age, language spoken at home and history of migration into Germany were asked as demographic variables (Mang et al., 2018; German Statistisches Bundeamt (2021)).

### 3.6. Statistical analyses

For the randomization check, we used a multivariate general linear model (MANCOVA) with the five field trip days as independent variables and the five personality dimensions as dependent variables. Further trait variables that were measured prior to the field trip (need for cognition, birding specialization, bird species knowledge, age) were compared with a univariate linear model. To compare the gender distribution across the five field trip days, we used a chi-square test. We used Pearson’s correlation analysis for the relationship between the psychological variables. In addition, significant predictor variables were entered in a backward elimination procedure in a linear regression to assess the most important predictor variables of recalled restoration and self-reported restoration. Spearman rho correlations were used to relate bird species richness to psychological restoration. This non-parametric test-design was needed as parametric test assumptions, such as normality (e.g., bird species richness showed a non-normal distribution) were not fulfilled. SPSS 28 was used for all analyses.

## 4. Results

First, a randomization check was carried out whether the participants of the five field trips differed in their personality. The randomization check showed that the five personality dimensions were evenly distributed across the five field trip days, i.e., there were no differences in the personality of the participants at the different field trips (Wilk’s *λ* = 0.901, *F* = 0.646, *p* = 0.877). Further, there were no differences among the participants across the five field trip days in birding specialization (*F* = 1.045, *p* = 0.387), bird-related interest (*F* = 1.195, *p* = 0.316), prior knowledge (*F* = 0.186, *p* = 0.945), age (*F* = 0.505, *p* = 0.732) or need for cognition (*F* = 1.544, *p* = 0.194). Genders were evenly distributed across the field trips (*χ*^2^ = 5.824, *df* = 8, *p* = 0.667). This study uses bivariate analyses to examine relationships with restoration (Table 1). We found that bird species knowledge and birding specialization had no influence on psychological restoration. General bird-related interest did not correlate with the restoration outcomes. Need for cognition correlated with psychological restoration, suggesting that curious people and interested people benefit more from the restoration. Concerning intrinsic motivation, the three scales enjoyment, perceived competence, and perceived choice were positively correlated with restoration, but not pressure/tension. Regarding the learning emotions interest and well-being were positively related to restoration, while boredom was negatively related. Thus, high well-being and interest and low boredom are positively influencing restoration.

**Table 1:** Predictors of the dimensions recalled restoration and psychological restoration.

		Recalled restoration	Psychological restoration
Bird knowledge test (prior to bird walk)	*r*	−0.142	−0.045
	*p*	0.105	0.611
Bird-related interest	*r*	0.103	0.169
	*p*	0.241	0.053
Birding specialization	*r*	−0.063	0.103
	*p*	0.472	0.238
Intrinsic motivation
Enjoyment	*r*	0.382^***^	0.325^***^
	*p*	<0.001	<0.001
Perceived choice	*r*	−0.008	0.198^*^
	*p*	0.929	0.023
Perceived competence	*r*	0.039	0.179^*^
	*p*	0.660	0.040
Pressure/tension	*r*	−0.032	−0.144
	*p*	0.712	0.100
Learning emotions
Well-being	*r*	0.427^***^	0.295^***^
	*p*	<0.001	<0.001
Boredom	*r*	−0.419^***^	−0.284^***^
	*p*	<0.001	<0.001
Interest	*r*	0.248^**^	0.267^**^
	*p*	0.004	0.002
Big five personality
Conscientiousness	*r*	0.129	0.061
	*p*	0.144	0.490
Openness	*r*	0.098	−0.028
	*p*	0.262	0.749
Agreeableness	*r*	0.090	0.164
	*p*	0.307	0.061
Extraversion	*r*	0.050	0.064
	*p*	0.568	0.467
Neuroticism	*r*	0.029	−0.099
	*p*	0.742	0.259
Need for cognition	*r*	0.261^**^	0.246^**^
	*p*	0.003	0.004
Age (year of birth)	*r*	0.109	0.111
	*p*	0.212	0.205

^*^p < 0.05, ^**^p < 0.01, ^***^p < 0.001, *N* = 132.

Bivariate relationships between recalled restoration and psychological restoration with birding specialization, bird-related interest, bird knowledge test, intrinsic motivation, personality, need for cognition and demographics (age) measures. Spearman rho rank correlations were applied.

**Figure 1:**
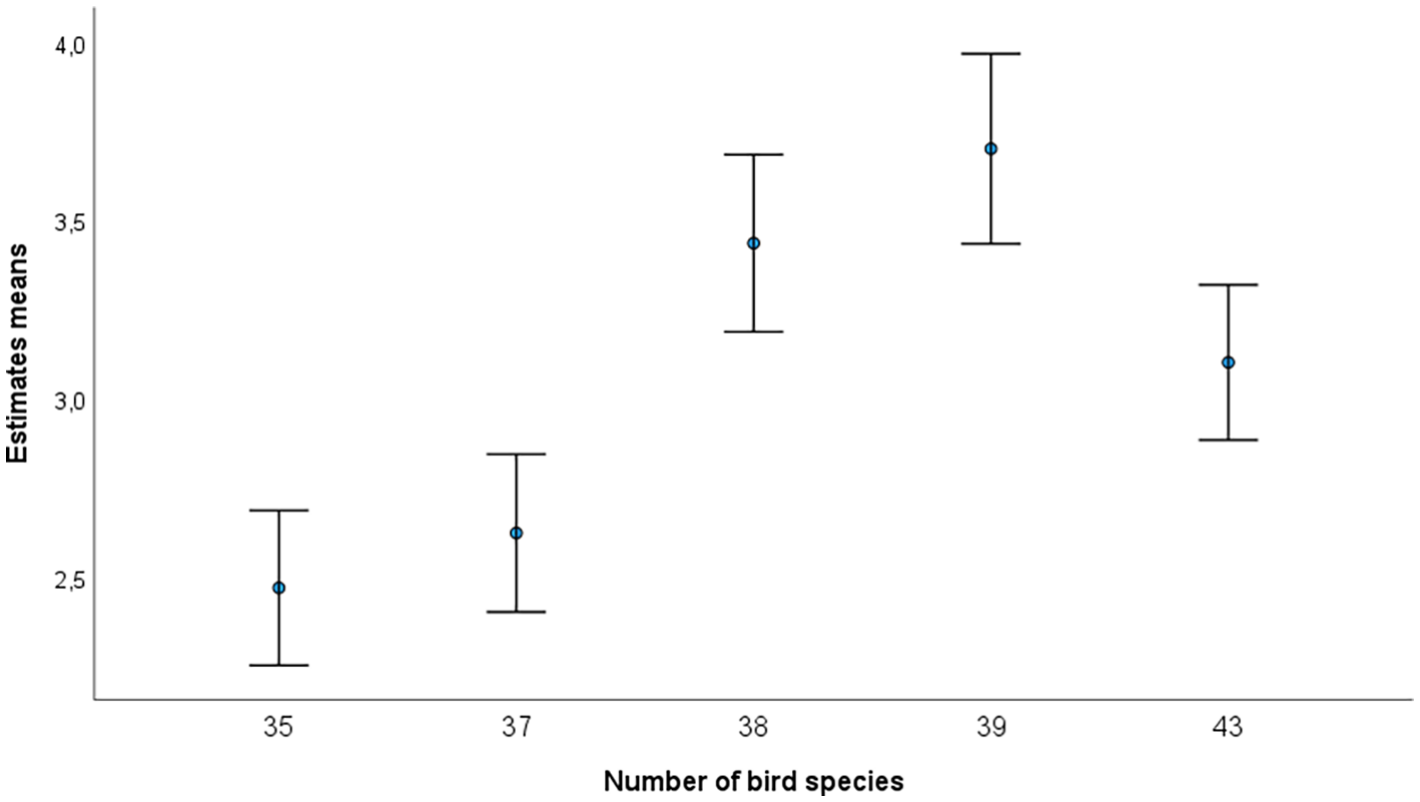
Bird species richness and recalled restoration. Relationship between recalled restoration (note scale ranges from 1 = “less rested than before,” “neither more nor less rested,” “a little more rested,” “more rested,” to 5 = “much more rested” than before) and number of bird species recorded at each of the five field trip days. Means and one standard error are given.

Concerning demographic variables, we found no differences between genders, languages spoken at home, and migration history (*p* > 0.10). Age did not correlate with any of the outcome variables (Table 1). All five dimensions of personality were unrelated to the restorative outcome. Birding specialization was correlated with bird interest (*r* = 0.568, *p* < 0.001) and the knowledge test (prior to the bird walk: *r* = 0.759, *p* < 0.001). Bird interest, in turn, was related to bird knowledge test scores (prior to the bird walk: *r* = 0.506, *p* < 0.001). We entered all significant variables from the bivariate correlations into a linear regression in a backward elimination procedure. Recalled restoration was predicted by boredom (*β* = −0.381, *T* = −4.757, *p* < 0.001) and need for cognition (*β* = 0.183, *T* = 2.279, *p* = 0.024). Thus, participants feeling less bored and with a higher need for cognition experienced a higher restoration (full model: *F*_2,129_ = 16.849, *p* < 0.001, corrected *R*^2^ = 0.195). Concerning psychological restoration, the predictors were enjoyment (*β* = 0.244, *T* = 2.840, *p* = 0.005), perceived choice (*β* = 0.166, *T* = 1.999, *p* = 0.048) and need for cognition (*β* = 0.190, *T* = 2.238, *p* = 0.027; full model: *F*_3,128_ = 8.060, *p* < 0.001, corrected *R*^2^ = 0.139). There was a significant relationship between the number of bird species present during the field trips and recalled restoration (*r_s_* = 0.259, *p* < 0.003; Figure 1), but not between species number and psychological restoration (*r_s_* = 0.104, *p* = 0.237).

## 5. Discussion

This study examines the impact of a birding activity on psychological restoration. Our results are encouraging because participants psychological restoration was independent of prior knowledge, personality, and demographic effects. This suggests that people can recover and restore irrespective of such unchangeable variables. However, we did find that need for cognition was related to restoration.

Recalled restoration was related to the number of bird species present on the respective day, which emphases and corresponds to studies pointing to the importance of bird species richness and biodiversity on health and well-being (Luck et al., 2011; Hepburn et al., 2020; Methorst et al., 2021). In addition to previous studies (Fuller et al., 2007; Dallimer et al., 2012), we collected bird data in a situational manner, i.e., the bird species richness could be directly related to the field trip. In combination with the fact that bird species knowledge was unrelated to the restoration outcome measures, our study is another brick stone of evidence for the Attention Restoration Theory (Kaplan and Kaplan, 1989). Kaplan and Kaplan (1989) suggested that psychological restoration is an unconscious fact, and that people can restore their attention even with a low knowledge of their surrounding biodiversity. Moreover, in addition to bird species knowledge, birding specialization and bird-related interest were unrelated to the restorative outcomes. This means that participants with low interest in birds or for whom birding is hardly or not at all a leisure activity benefited in the same way as participants with high interest and higher leisure orientation towards birding. This is a highly encouraging result and fits the previously published results Randler et al. (2022a). However, in their previous study Randler et al. (2022a), specifically studied leisure birdwatchers, while in this current one, almost all participants were novices. Also, restoration was asked for in a situational manner, i.e., directly related to the actual event, while it was operationalized on a trait level by Randler et al. (2022a) to gain a general perspective on birding and well-being. Our current data are further encouraging because they show that interest is not necessary for psychological restoration, and even more, that a higher interest does not automatically lead to a higher restoration. As people can perceive restoration without any substantial bird knowledge, a guided field trip can also have a therapeutic function (Tryjanowski et al., 2022).

Previous studies reported inconclusive relationships between personality and psychological restoration and found no clear and consistent effect. Senese et al. (2020) reported a weak and negative association between extraversion and the change scores for fascination and being-away during a virtual reality experience. Further, lower levels of neuroticism extracted higher psychological benefits from greenspaces (Ambrey and Cartlidge, 2017). These results are inconclusive, but also point to a need for further studies. Notably, personality may have an impact on psychological restoration, but may be dependent on demographics, context, and situational factors. In our dataset, we could not apply detailed sub-group analyses because the sample size was lower compared to the previous survey studies (Senese et al., 2020; Feng et al., 2022). In the future, a higher number of participants should be studied in such controlled experiments (see also Murawiec et al., 2021). The discrepancy of the results concerning the relationship between restoration and personality may be owed to the different measures of restoration (Ambrey and Cartlidge, 2017; Senese et al., 2020; Feng et al., 2022). Further, it may be related to the different study environments (virtual reality in Senese et al., 2020; survey studies in Feng et al., 2022 and Ambrey and Cartlidge, 2017). In the current study, we found no relationship between personality traits and restoration. However, our study differed from the others since we controlled for the environment of the restorative activity (guided bird walk), but Subiza-Pérez et al. (2021) also reported no relationship between the big five personality domains and restoration.

As a new predictive variable, we identified the need for cognition being related to psychological restoration. This was confirmed in the regression analyses. As the need for cognition is a trait characteristic, we have measured it prior to the guided bird walk. A causal relationship of the result can be inferred, meaning that the need for cognition has a positive impact on later restoration. We assume that participants with a high need for cognition were more curious (Cacioppo et al., 1996), and probably more prone and open to learning and experiencing new bird species. This has implications for designing restorative environments, because people with a higher need for cognition may prefer environments that give them an adequate stimulus that may be higher in comparison with others that score lower on the need for cognition. In detail, this means that an average environment with new stimuli, but not too stimulative fits peoples’ restorative needs best (Kaplan and Kaplan, 1989). But still, individuals may differ in their need for cognition, and hence, in the optimal stimuli needed for restoration.

Intrinsic motivation has been rarely addressed in the context of psychological restoration. However, perceived competence was found to matter in a restorative environment for gardeners (Kaplan and Kaplan, 2009), thus experiencing competence may produce restoration. Learning motivations were related to psychological restoration in the same manner as they have been related to academic achievement and educational outcomes (Gläser-Zikuda et al., 2005; Wilde et al., 2009; Randler et al., 2011). Thus, well-being and interest were positively, and boredom, in turn, negatively related to restoration. To our knowledge, this has not been addressed before, because previous studies based on surveys did mostly not include educational programs (exception: Murawiec et al., 2021).

### 5.1. Policy implications

Our study suggests adding restorative function variables of more cognitive-oriented programs in subsequent studies because people also may have a need for cognition when it comes to restoration. Similarly, this kind of novelty interest is found in tourism (Chark et al., 2020). We also suggest a broader focus on education and cognitive aspects when it comes to linking biodiversity and health within the frame of ecosystem services.

### 5.2. Limitations

All field trips were led by the same persons in the same area, so future studies might be scaled up by studying different field trip leaders in diverse urban green and blue spaces. Further studies might also apply a comparison group without birding field trips (e.g., a walking activity) to disentangle possible influences of learning and the need for cognition. This group may just walk around the area without watching birds and receiving information. Additionally, the small sample size only allowed for limited analysis, with a larger data set, more advanced methodologies could be applied, for example examining subgroup-effects and potential moderating/mediating variables. The linear regressions should therefore be treated with caution. We strongly encourage further studies with a different focus on physical restoration and measuring physiological variables (e.g., Lindemann-Matthies and Matthies, 2018). In addition, perceived bird diversity should be assessed in the future to compare it with the measured diversity. Nevertheless, some previous studies reported that lay people may be able to roughly estimate the bird diversity in the environment (Vanhöfen et al., 2022).

## 6. Conclusion

In conclusion, lower-pace birding seems a good restorative activity for many people, irrespective of their personality, knowledge, and prior interest. Our data support the suggestions of Kaplan and Kaplan (2009) who advised to collaborate with other disciplines within and outside psychology, thus, educational psychology and biology/ecology may contribute to advancing the nature—human relationship. Further, studying individual traits, like personality traits and other aspects, such as curiosity may further advance the field. Also, as personality is a trait measure and restoration was assessed on a situational (state) level, we may infer causal relations meaning that personality in our study is not a predictor of restoration. We strongly suggest future research to include differential aspects when analyzing psychological restoration, depending on the restorative environment. In our case, we suggest using the need for cognition in relationship with restoration when it has at least to some extent a cognitive component, like in the guided bird walk.

## Data availability statement

The raw data supporting the conclusions of this article will be made available by the authors, without undue reservation.

## Ethics statement

The studies involving human participants (ID: A2.5.4-225_aa) were reviewed and approved by the ethics committee of the Faculty of Social Sciences and Economics of the University of Tübingen. Date of ethics committee approval: June 14, 2022. The patients/participants provided their written informed consent to participate in this study.

## Author contributions

CR, CF, JV, TH, CE, FN: conceptualization, data curation, methodology, validation, writing—review and editing. CR and JV: formal analysis. CF and CR: funding acquisition, project administration, and resources. CR, JV, TH, CE, and FN: investigation. CF, CR, JV, TH, and CE: software. CF, supervision. CR, JV, and CF: roles/writing—original draft. All authors contributed to the article and approved the submitted version.

## Funding

This research is supported by the LEAD Graduate School and Research Network (GSC1028), which was funded within the framework of the Excellence Initiative of the German federal and state governments. We acknowledge support by Open Access Publishing Fund of University of Tübingen.

## Conflict of interest

The authors declare that the research was conducted in the absence of any commercial or financial relationships that could be construed as a potential conflict of interest.

## Publisher’s note

All claims expressed in this article are solely those of the authors and do not necessarily represent those of their affiliated organizations, or those of the publisher, the editors and the reviewers. Any product that may be evaluated in this article, or claim that may be made by its manufacturer, is not guaranteed or endorsed by the publisher.

## Footnotes

^1^ https://ebird.org/hotspot/L10365184

^1^ https://www.dlr.rlp.de/Agrarmeteorologie-BW/Wetterdaten/Stationen-nach-Region/Tuebingen/BWAM146

## Appendix 1

eBird checklist of the different field trips:

eBird Checkliste—20 Jul 2022—Hirschauer Baggersee—35 Arten.

eBird Checkliste—19 Jul 2022—Hirschauer Baggersee—38 Arten.

eBird Checkliste—13 Jul 2022—Hirschauer Baggersee—43 Arten.

eBird Checkliste—12 Jul 2022—Hirschauer Baggersee—39 Arten.

eBird Checkliste—6 Jul 2022—Hirschauer Baggersee—37 Arten.
